# Fusion of contrast-enhanced magnetic resonance neurography and T1-weighted imaging improves simultaneous nerve-tumor visualization in head and neck lesions

**DOI:** 10.3389/fonc.2025.1692893

**Published:** 2025-10-22

**Authors:** Tingting Liu, Zhiqing Zhang, Yuan Liu, Yu Su, Qun Yu, Chungao Li, Xiangchuang Kong, Jie Zhao, Shuo Huang, Chuansheng Zheng, Wenjun Wu, Lixia Wang

**Affiliations:** ^1^ Department of Radiology, Union Hospital, Tongji Medical College, Huazhong University of Science and Technology, Wuhan, China; ^2^ Hubei Province Key Laboratory of Molecular Imaging, Wuhan, China; ^3^ Hubei Provincial Clinical Research Center for Precision Radiology & Interventional Medicine, Wuhan, China

**Keywords:** contrast-enhanced magnetic resonance neuroimaging, peripheral nerve tumors, image fusion technology, neurogenic tumors, non-neurogenic tumors

## Abstract

**Objective:**

To investigate the application value of integrating contrast-enhanced magnetic resonance neurography (CE-MRN) with contrast-enhanced T1-weighted imaging (CE-T1WI) to improve the simultaneous imaging of nerves and tumors in the head and neck.

**Materials and methods:**

A retrospective study of 31 patients (14 neurogenic, 17 non-neurogenic) with pathologically confirmed peripheral nerve tumors (2017–2024) was conducted. All underwent 3.0 T MRI, assessed by two blinded radiologists. Tumor involvement patterns, enhancement features, MRI signs, and normalized nerve signal intensity were analyzed. Diagnostic confidence and lesion conspicuity were compared across CE-MRN, CE-T1WI, and fusion images. Statistical analysis included Mann-Whitney U test and interobserver agreement (Kappa/ICC).

**Results:**

Interobserver agreement was moderate to excellent (Kappa/ICC: 0.47-0.93). Focal involvement dominated in neurogenic tumors (92.9% vs. 52.9% diffuse in non-neurogenic, p=0.002). Traditional MRI signs: “dumbbell sign” was more frequent in neurogenic tumors (78.6% vs. 11.8%, p<0.001), while “effacement of fat plane” was common in non-neurogenic (70.6% vs. 0%, p<0.001). Novel CE-MRN signs: “enhanced target sign” (28.6% vs. 0%, p=0.032) and “nerve tail sign” (57.1% vs. 11.8%, p=0.018) were neurogenic markers, whereas “nerve effacing sign” was non-neurogenic (76.5% vs. 35.7%, p=0.033). Affected nerves showed higher signal intensity than contralateral nerves (p<0.05). Fusion images matched CE-MRN in diagnostic confidence and surpassed CE-T1WI in conspicuity (p<0.001).

**Conclusion:**

Image fusion technology addressed the limitations of CE-MRN in lesion visualization, thereby enhancing diagnostic confidence. The novel signs and nerve signal alterations observed in CE-MRN provide visual evidence for the accurate diagnosis and differentiation of head and neck tumors.

## Introduction

The accurate diagnosis and differentiation of peripheral nerve tumors in the head and neck region represent a significant challenge within the field of clinical neuroimaging. Primary neurogenic tumors, included schwannomas and neurofibromas, as well as head and neck malignancies, such as nasopharyngeal carcinoma, adenocystic carcinoma and lymphoma, which frequently involve peripheral nerves, demonstrate marked differences in biological behavior, treatment strategies, and prognostic outcomes. Among the secondary peripheral nerve tumors, perineural invasion (PNI) and perineural spread (PNS) serve as key pathological phenotypes for malignant tumors invading nerves, which are strongly associated with unfavorable prognosis (1, 2). Approximately 30%–40% of patients have already progressed to the advanced stage by the time neurological symptoms become apparent. A missed diagnosis of nerve involvement places patients at a higher risk of recurrence and poorer prognosis (3, 4). Although histological examination serves as the gold standard for PNI and PNS, pretreatment detection of nerve involvement still predominantly rely on imaging techniques. Consequently, there is an urgent requirement for a highly sensitive and specific non-invasive visualization method to fulfill unmet clinical needs.

In the domain of neuroimaging, conventional imaging techniques possess distinct clinical significance while also presenting opportunities for improvement. Ultrasound enables dynamic observation of nerve bundle structures; however, their penetration depth restrict their application in deep nerve imaging (5). CT can depict bone structure involvement, yet their soft tissue contrast is suboptimal. 18F-FDG PET/CT is helpful for detecting abnormal glucose metabolism in nerve damage regions and aiding disease classification, but its spatial resolution is insufficient to visualizing neural structures (6). Conventional MRI, characterized by its superior soft tissue contrast and spatial resolution, has emerged as the primary imaging modality for tumor imaging (7). However, it still lacks the capability to directly visualize nerves. MRI diagnosis of PNI/PNS often depends on indirect signs, including the occupying and blurring of the perineural fat plane, as well as abnormal enhancement along nerve regions, which may be influenced by partial volume effects and the expertise level of the clinicians. Recently, the advancement of magnetic resonance neurography (MRN), particularly contrast-enhanced magnetic resonance neurography (CE-MRN), has substantially improved the visualization of peripheral nerves and their small branches. This development has also introduced novel indicators for assessing nerve involvement, potentially enabling direct detection of peripheral nerve tumors (7–10).

Previous research has largely focused on one imaging method, but multisequence image fusion and rendering technologies have provided new perspectives for comprehensive evaluation on neuro-oncological conditions. Ensle F et al. found that fusion of DESS and enhanced STIR sequences enables the preservation of both spatial details and tissue contrast derived from the characteristics of both sequence, thereby providing optimal nerve visualization (11). Xu Z et al. integrated MRA and MRN images, revealing the anatomical relationship between brachial plexus neuropathy and associated vascular abnormalities, thus providing multi-dimensional imaging evidence for clinical decision-making in treatment (12). He A et al. applied heatmap color rendering technology in MRN, which substantially enhanced the visualization of lumbosacral plexus neuropathy. This advancement particularly bolstered diagnostic confidence among less experienced physicians and effectively reduced inter-observer variability in subjective assessment (13). These advancements provided valuable insights for addressing the limitation of CE-MRI in poorly displaying the contour of enhanced tumor parenchyma, as enhancement shows diffused decreased signal intensity on CE-MRI images (14).

Therefore, we hypothesize that image fusion technology of CE-MRN and contrast-enhanced T1-weighted imaging(CE-T1WI) will enhance the identification of nerve involvement by tumors. This study aims to compare the differences in classical MRI signs and novel CE-MRN signs, as well as nerve signal changes between neurogenic and non-neurogenic tumors. Additionally, this study also seeks to evaluate whether fusion images provide superior performance compared to single-sequence images in improving diagnostic confidence and lesion conspicuity for head and neck tumors.

## Materials and methods

### Subject

**Figure 1:**
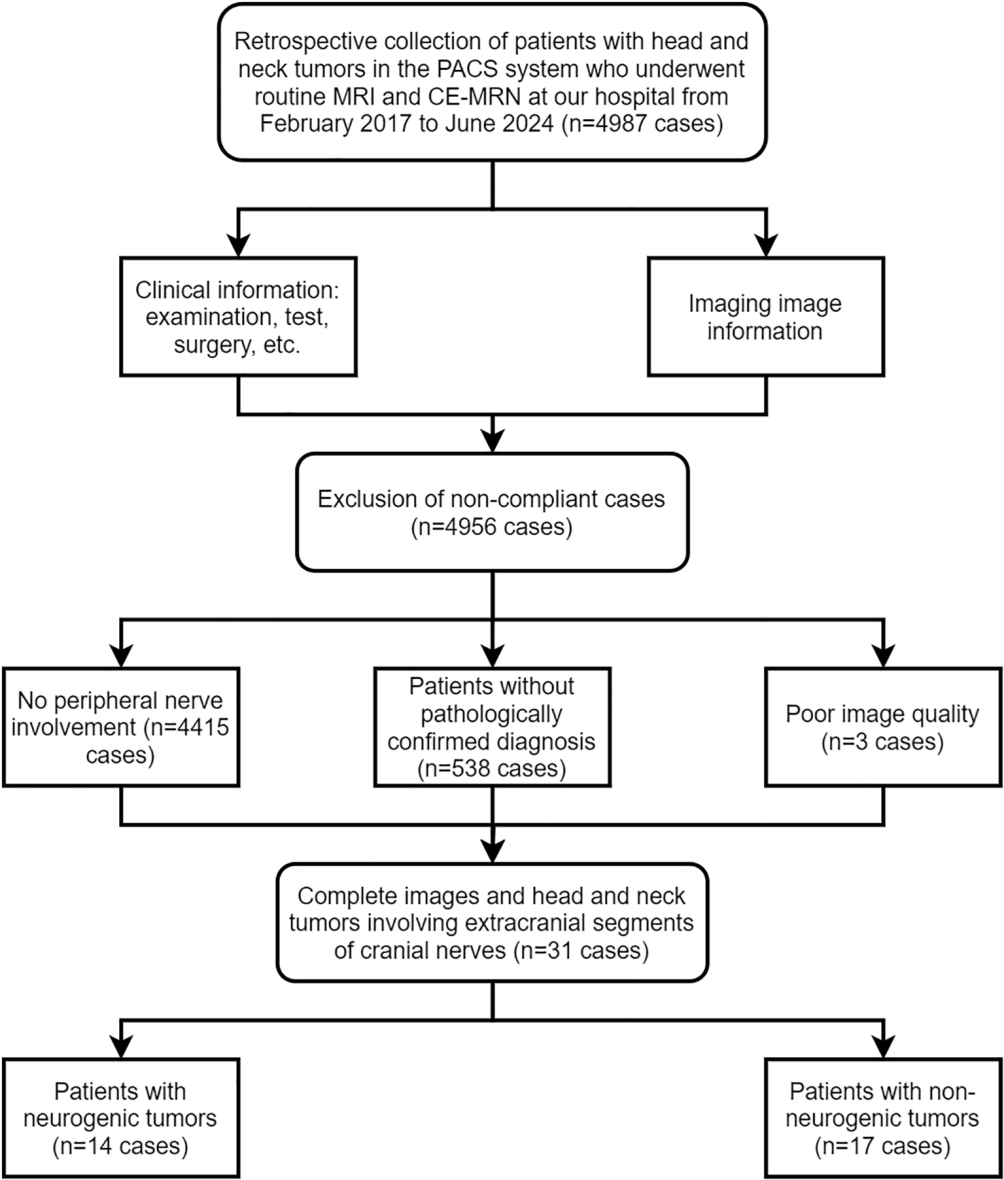
Flowchart.

This study was a retrospective study conducted in accordance with the principles of the Declaration of Helsinki and approved by the Ethics Committee of our hospital (UHCT241287), informed consent from patients was waived. The Pathology Report System and Picture Archiving and Communication System (PACS, Carestream, Shanghai, China) was researched between February 2017 and June 2024. Inclusion criteria included: 1) patients who underwent both routine MRI and CE-MRN examinations; 2) imaging reports indicating space-occupying lesions involving peripheral nerves; 3) pathological confirmation obtained via biopsy or surgery. Exclusion criteria included: 1) images that could not be reconstructed for evaluation due to severe artifacts; 2) incomplete imaging data. A total of 4,987 patients who underwent CE-MRN examinations were screened. Patients without reported peripheral nerve involvement (n=4,415), without pathological confirmation (n=538), and with poor image quality (n=3) were excluded. Ultimately, 31 patients with complete imaging data and confirmed peripheral nerve tumors were included. Patients were divided into two groups based on pathological results: the neurogenic tumor group (n=14) and the non-neurogenic tumor group involving peripheral nerves (n=17) (**Figure 1**).

### MRI imaging protocol

All examinations were performed using 3.0 T MRI systems (Philips Ingenia CX, Best, Netherlands, or Siemens Healthcare, Skyra, Erlangen, Germany). Head and neck nerve imaging was performed using a 20-channel head and neck combined coil. The MRI protocol included: axial T1-weighted imaging (T1WI), axial T2-weighted imaging (T2WI), axial T2 FLAIR, axial CE-T1WI, coronal CE-T1WI, and coronal CE-MRN (Philips: 3D NerveView sequence; Siemens: T2 STIR SPACE sequence). The contrast agent (Magnevist; Bayer AG, Leverkusen, Germany) was administered via an intravenous catheter at a flow rate of 1.5 mL/s (dose: 0.3 mL/kg), followed by 15 mL of saline at the same rate. The CE-MRN sequence was initiated 1–1.5 minutes post-injection. The specific parameters for CE-T1WI and CE-MRN are outlined as **Table 1**.

**Table 1:** Imaging parameters of coronal CE-T1WI and CE-MRN.

MRI system		TR/TE (ms)	TI (ms)	FOV (mm^2^)	Matrix	ETL	Voxel size (mm^3^)	Average	Acquisition time
Siemens	coronal CE-T1WI	5.54/2.46	–	272×340	320×320	–	0.9×0.8×1.0	1	1:56
	coronal CE-MRN	3000/287	220	190×190	218×256	150	0.4×0.4×1.0	2	8:03
Philips	coronal CE-T1WI	4.1/1.49	–	300×243	300×242	40	1.00×1.00×2.00	2	1:09
	coronal CE-MRN	2000/230	260	250×199	312×247	50	0.80×0.81×2.00	2	6:30

TR, repetition time; TE, echo time; TI, inversion time; FOV, field of view; ETL, echo train length.

### MRI imaging assessment

The initial qualitative assessment and basic measurements on the selected images were performed by two radiologists with 8 and 3 years of neuroimaging experience, respectively, under blinded conditions using RadiAnt DICOM Viewer (Medixant, Poland, version 5). Three-dimensional images were reconstructed using maximum intensity projection (MIP) and multi-planar reconstruction (MPR). The assessment included: patterns of involved nerves (mono nerve/multiple nerves; focal/multifocal/diffuse), enhancement (none, homogeneous, heterogeneous, periphery); nerve morphological and signal changes (hypertrophy or atrophy, signal increase or decrease); classical MRI signs (target sign, tail sign, dumbbell sign, effacemant of fat space); and novel CE-MRN signs defined in a previous study (enhanced target sign, nerve effacing sign, nerve compressing sign, nerve tail sign) (14). The final results were determined through a consensus reached by two radiologists.

### Quantification of nerve signal intensity

In the CE-MRN sequence, both neurogenic and non-neurogenic tumors showed increased signal intensity in some distal nerves. Two trained neuroradiologists measured the signal intensity of the distal nerves on both affected and contralateral side. Then the nerve signal intensity was normalized relative to the signal of adjacent muscles. The signal intensity ratio of nerve-to-muscle (SI_nerve/muscle_) was compared the involved distal nerves and the contralateral uninvolved nerves.

### Assessment of diagnostic confidence and lesion visibility

The ITK-SNAP toolkit (ITK, Insight Segmentation Toolkit, version 3.8.0) was used to fuse coronal CE-MRN and CE-T1WI images. The images were first co-registered using rigid or affine transformation. Subsequently, the CE-T1WI was set as the base image, and the CE-MRN was imported in overlay mode. Optimal visual fusion was achieved by adjusting the opacity and applying a color map. Diagnostic confidence and lesion conspicuity were evaluated using a 4-point scale. The diagnostic confidence scoring criteria were as follows: 0 = unable to determine the relationship between the lesion and the nerve; 1 = low confidence (suggesting an uncertain inference of the relationship); 2 = moderate confidence (indicating a probable inference of the relationship); 3 = high confidence (clear visualization of the nerve and explicit delineation of its relationship with the lesion). The assessment of lesion conspicuity was conducted by evaluating the clarity of the lesion contour and used the following scoring criteria: 0 = unable to discern the lesion contour; 1 = less than 50% of the lesion contour is visible; 2 = more than 50% of the lesion contour is visible; 3 = the complete lesion contour can be fully determined.

### Statistical analysis

All data were analyzed using SPSS (version 25.0; IBM Corp.; Chicago, IL, USA). The normality of continuous data between the neurogenic and non-neurogenic tumor groups was tested using the Shapiro-Wilk (S-W) method. Data with normal distribution were expressed as mean ± standard deviation (Mean ± SD). For non-normally distributed data, the median (interquartile range) was used. Categorical variables were compared using the chi-square test or Fisher’s exact probability test. Intergroup consistency was assessed using Cohen’s Kappa (for qualitative data) and intraclass correlation coefficient (ICC, for quantitative data), with the following criteria: 0.81–1.00: excellent, 0.61–0.80: good, 0.41–0.60: moderate, 0.21–0.40: fair, 0.00–0.20: poor. Intergroup comparisons of diagnostic confidence and lesion conspicuity scores (between CE-T1WI and CE-MRN, between CE-T1WI and fused images, and between CE-MRN and fused images) were performed using the Mann-Whitney U test. Multiple comparisons were adjusted using the Bonferroni method, with p-values automatically adjusted. A p-value < 0.05 was considered statistically significant.

## Results

### Demographic and clinical characteristics

There were no significant differences between the neurogenic tumor group and the non-neurogenic tumor group in terms of mean age, gender, or number of involved nerves (p > 0.05 for all). Tumors in the neurogenic group were predominantly focal lesions (92.9%), whereas those in the non-neurogenic group were predominantly diffuse (52.9%), with a significant difference (p = 0.002). More detailed information can be found in **Table 2**.

**Table 2:** Demographic and clinical characteristics.

Baseline data	Neurogenic tumors(n=14)	Non-neurogenic tumors involving nerves(n=17)	P value	Kappa
Gender, n (%)			0.289	–
M	8 (57.1%)	6 (35.3%)		
F	6 (42.9%)	11 (64.7%)		
Age (year)	55.214 ± 13.291	56.588 ± 11.938	0.764	–
Involved nerve, n (%)			0.477	0.865
Mono nerve	6 (42.9%)	5 (29.4%)		
Multiple nerves	8 (57.1%)	12 (70.6%)		
Lesion pattern, n			0.002*	0.927
Focal	13 (92.9%)	8 (47.1%)		
multifocal	1 (7.1%)	0 (0%)		
Diffuse	0 (0%)	9 (52.9%)		
Pathological Diagnosis	schwannoma (n=15) Neurofibroma (n=2)	nasopharyngeal carcinoma (10) adenoid cystic carcinoma (n=2) Oropharyngeal Carcinoma (n=1) Sinonasal Carcinoma (n=1)		

### Inter-reader agreement

In terms of MRI signs identification, the Kappa values for conventional MRI signs ranged from 0.47 to 0.93 (moderate to excellent); for CE-MRN signs, the Kappa values ranged from 0.65 to 1.00 (good to excellent). Regarding inter-reader agreement in diagnostic confidence scores, the Kappa value for CE-T1WI was 0.54 (moderate), and 0.77 (good). for CE-MRN. Inter-reader consistency for lesion conspicuity scores showed Kappa values of 0.77 (good) for CE-T1WI and 0.77 (good) for CE-MRN. The highest consistency was observed for diagnostic confidence (Kappa = 0.89) and lesion visibility (Kappa = 0.87) in fused images. Additionally, the inter-reader reliability in nerve signal measurements was good (ICC > 0.800).Observers showed good to excellent agreement on the findings across different models of MR scanners (ICC > 0.765) (**Supplementary Table S1**).

### Comparison of MRI manifestations between neurogenic and non-neurogenic groups

**Figure 2:**
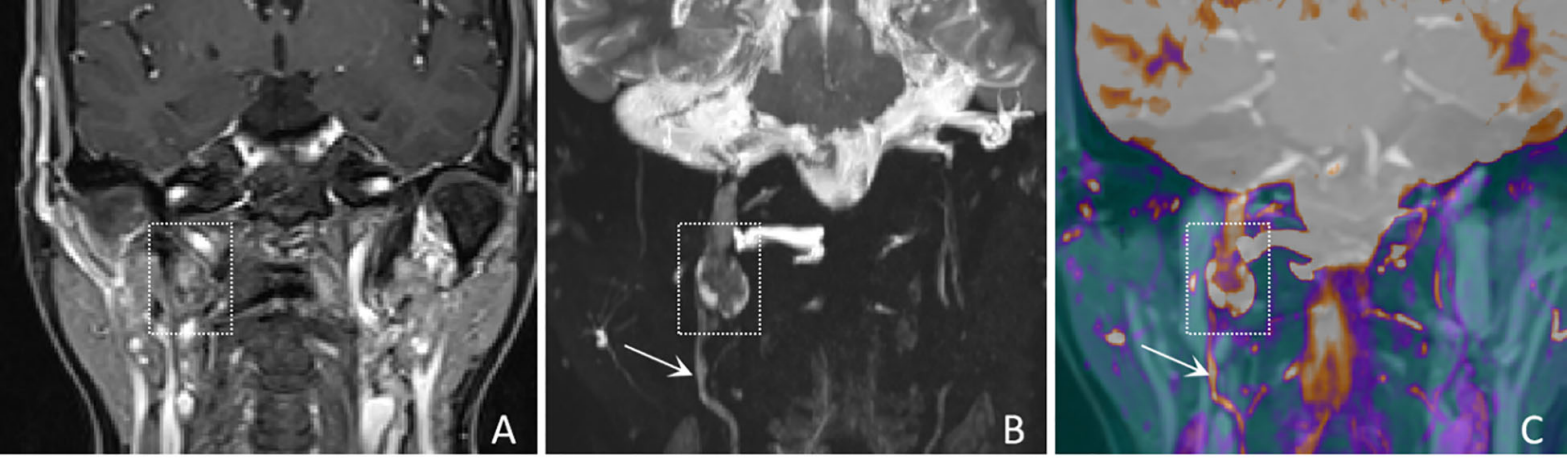
A 61-years-old female with a schwannoma of the right glossopharyngeal nerve, showing “enhanced target sign” and “nerve tail sign”. **(A)** A coronal CE-T1WI image shows a nodule inferior to the right jugular foramen, exhibiting heterogeneous enhancement (white dashed boxes), with complete obscuration of the nerve. **(B)** A coronal CE-MRN image shows a central low signal intensity and peripheral high signal intensity, referred to as the “enhancement target sign” (white dashed box) and “nerve tail sign” (white arrow marked). **(C)** A coronal fused image more clearly shows the lesion (white dashed box) and the involved nerve (white arrow).

**Figure 3:**
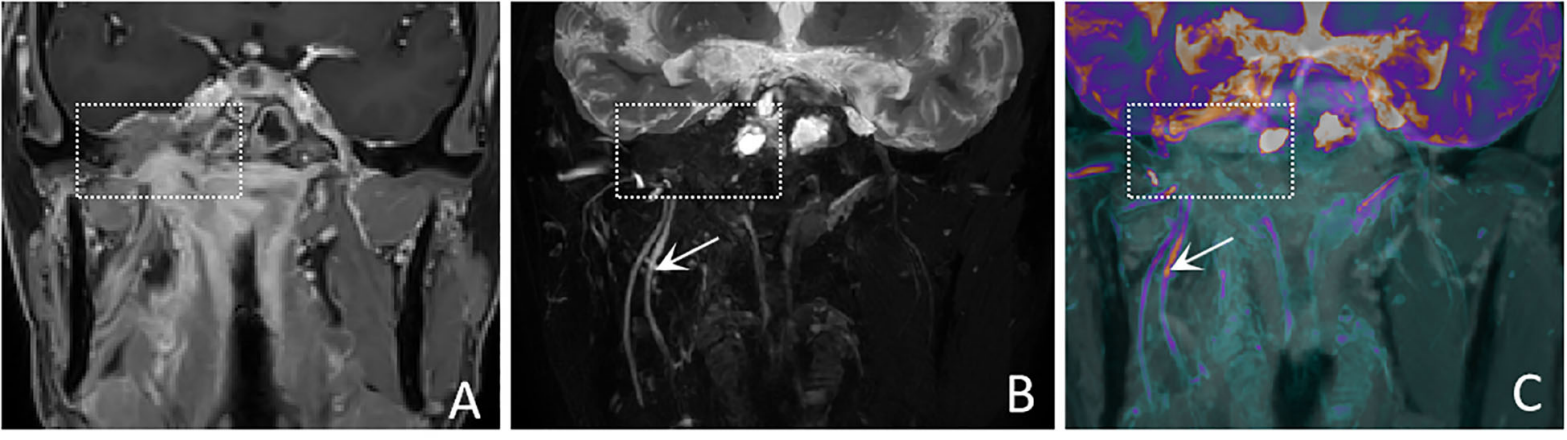
A 50-years-old male with nasopharyngeal carcinoma involving the right trigeminal nerve presents with the “nerve effacing sign”. **(A)** A Coronal CE-T1WI scan demonstrates diffuse enhancement in the parapharyngeal space and muscles, with invasion into the right side of the middle cranial fossa. (white dashed boxes) However, the trigeminal nerve and its branches are not visualized. **(B)** A coronal CE-MRN image shows diffuse signal decrease in the region of the mandibular nerve., indicative of the “nerve effacing sign” (white dashed box). Additionally, the right inferior alveolar nerve and lingual nerve are thickened and exhibit significantly increased signal intensity (white arrows). **(C)** A coronal fused image simultaneously displays the lesion (white dashed box) as well as the involved trigeminal nerve and its branches (white arrows).

In terms of nerve involvement pattern, the neurogenic group predominantly exhibited focal involvement (92.9%), while the non-neurogenic group predominantly exhibited diffuse involvement (52.9%), with a statistically significant difference (p=0.002). Classical MRI findings showed a significantly higher incidence of the “dumbbell sign” in the neurogenic group compared to the non-neurogenic group (78.6% vs. 11.8%, p < 0.001). The “effacement of the fat plane” was exclusively observed in the non-neurogenic group, albeit with a relatively lower incidence (29.4%) (p < 0.001)(**Table 3**). Among the novel CE-MRN signs, the “enhanced target sign” (p=0.032) and “nerve tail sign” (p=0.018) were more common in the neurogenic group (**Figure 2**). Conversely, the incidence of the “nerve effacing sign” was more frequently observed in the non-neurogenic group (76.5% vs. 35.7%, p=0.033), which may indicate a malignant trend (**Figure 3**). No significant differences were noted between the neurogenic and non-neurogenic groups regarding enhancement patterns, nerve signal and morphology (**Table 4**).

**Table 3:** Comparison of the incidence of classical MRI signs between the neurogenic tumor group and the non-neurogenic tumor group.

Classical MRI signs	Neurogenic tumors(n=14)	Non-neurogenic tumors involving nerves(n=17)	*P* value	Kappa
Enhancement pattern, n (%)			0.476	0.792
periphery	2 (14.3%)	0 (0.0%)		
inhomogeneous	9 (64.3%)	13 (76.5%)		
homogeneous	3 (21.4%)	3 (17.6%)		
none	0 (0.0%)	1 (5.9%)		
Target sign, n (%)			0.452	0.475
no	13 (92.9%)	17 (100%)		
yes	1 (7.1%)	0 (0.0%)		
Tail sign, n (%)			0.196	0.466
no	12 (85.7%)	17 (100%)		
yes	2 (14.3%)	0 (0.0%)		
Dumbbell sign, n (%)			< 0.001*	0.865
yes	11 (78.6%)	2 (11.8%)		
no	3 (21.4%)	15 (88.2%)		
Effacement of fat plane, n (%)			< 0.001*	0.931
no	14 (100%)	5 (29.4%)		
yes	0 (0.0%)	12 (70.6%)		

**Table 4:** Comparison of the incidence of novel CE-MRN signs between the neurogenic tumor group and the non-neurogenic tumor group.

CE-MRN signs	Neurogenic tumors (n=14)	Non-neurogenic tumors involving nerves (n=17)	*P* value	Kappa/ICC
Enhanced target sign, n (%)			0.032*	0.870
no	10 (71.4%)	17 (100%)		
yes	4 (28.6%)	0 (0.0%)		
Nerve effacing sign, n (%)			0.033*	0.934
no	9 (64.3%)	4 (23.5%)		
yes	5 (35.7%)	13 (76.5%)		
Nerve compressing sign, n (%)			1.000	0.652
no	14 (100%)	16 (94.1%)		
yes	0 (0.0%)	1 (5.9%)		
Nerve Tail sign, n (%)			0.018*	0.852
yes	8 (57.1%)	2 (11.8%)		
no	6 (42.9%)	15 (88.2%)		
Nerve Signal increase, n (%)			0.698	1.000
yes	11 (78.6%)	12 (70.6%)		
no	3 (21.4%)	5 (29.4%)		
Nerve Signal decrease, n (%)			0.664	1.000
no	12 (85.7%)	13 (76.5%)		
yes	2 (14.3%)	4 (23.5%)		
Nerve hypertrophy, n (%)			1.000	0.936
no	7 (50%)	8 (47.1%)		
yes	7 (50%)	9 (52.9%)		
Nerve atrophy, n (%)			0.488	1.000
no	14 (100%)	15 (88.2%)		
yes	0 (0.0%)	2 (11.8%)		

### Comparison of signal intensity between affected nerves and unaffected nerves

**Figure 4:**
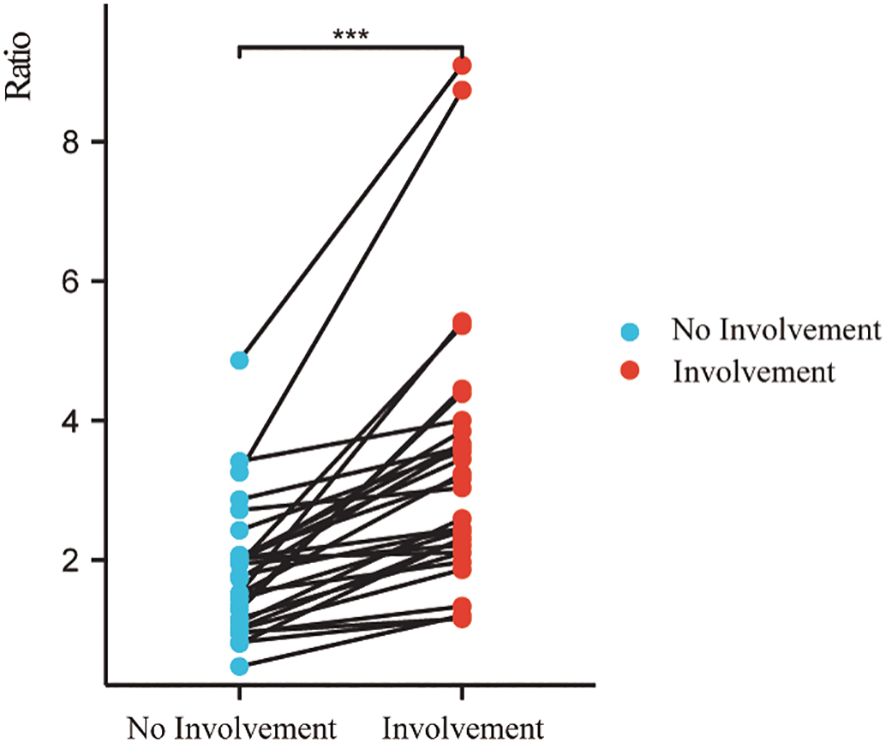
Signal intensity ratio of involved nerves was significantly higher than that of uninvolved nerves. (***p < 0.001).

The SI_nerve/muscle_ was significantly higher than that of contralateral uninvolved nerves/adjacent muscles (p < 0.001) (**Figure 4**).

### Comparison of diagnostic confidence and lesion conspicuity between CE-T1WI, CE-MRN, and fused imaging

**Figure 5:**
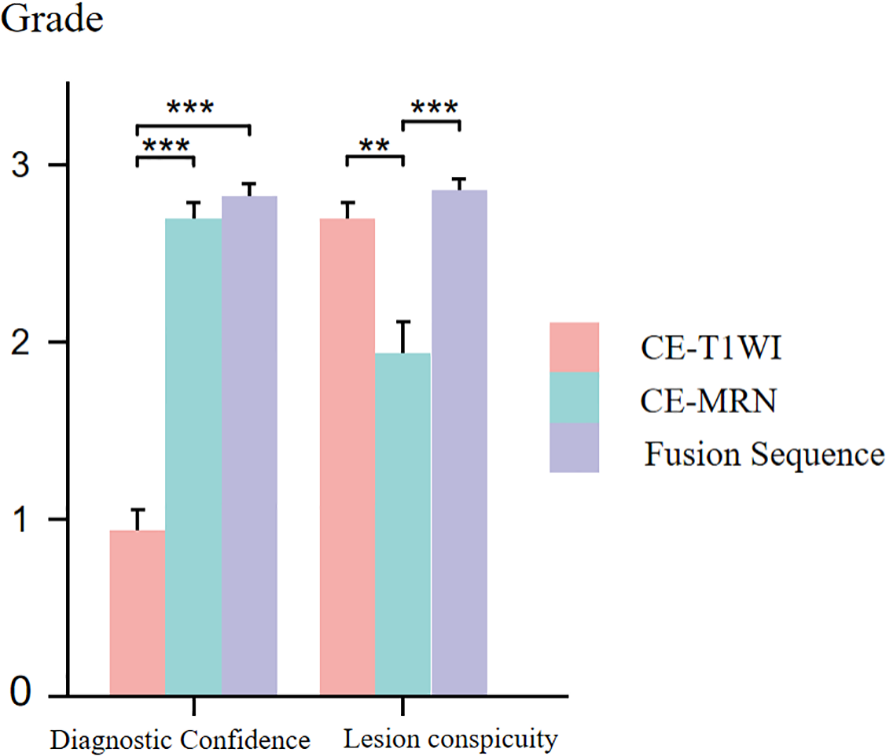
Comparison of diagnostic confidence and lesion visualization between CE-T1WI, CE-MRN and fused imaging. (**p < 0.01, ***p < 0.001).

In both neurogenic and non-neurogenic groups, as well as across all subjects, CE-MRN and fused imaging demonstrated significantly higher diagnostic confidence scores compared to CE-T1WI when identifying the relationship between lesions and nerves (p < 0.001). However, the lesion conspicuity scores of CE-MRN were markly lower than those of CE-T1WI and fused imaging (p < 0.01) (**Figure 5**).

## Discussion

CE-MRN demonstrates significant advantages in visualizing peripheral nerves but exhibits limitations in depicting solid tumor lesions. Present research concerning head and neck neuroimaging is chiefly concerned with dental surgery, nerve injury, or trauma and inflammatory conditions, and it predominantly uses 3D reconstruction instead of image fusion (15, 16). In this study, creatively using image fusion technology, we fused CE-T1WI and CE-MRN images to display both the enhanced tumor parenchyma and involved nerves, thereby achieving simultaneous imaging. This approach significantly improves diagnostic confidence for head and neck peripheral nerve tumors. Additionally, through a comparative analysis of the imaging characteristics between neurogenic and non-neurogenic tumors, we identified significant differences in nerve involvement patterns and MRI findings. We further elucidated the diagnostic value of both classical MRI signs and novel CE-MRN signs, including “enhanced target sign” “nerve tail sign” and “nerve effacing sign”, as well as nerve signal changes in distinguishing neurogenic and non-neurogenic tumors involving nerves in the head and neck region.

This study reveals that nerves are mainly affected focally in neurogenic tumors, whereas affected diffusely in the non-neurogenic malignancies. observation aligns with the localized expansive growth characteristics of benign neurogenic tumors such as schwannomas, and also reflects the pathological characteristics of malignant tumors spreading along the perineural spaces (17–19). In the novel CE-MRN signs (14), the “Enhanced target sign” manifests as a central region of low signal intensity surrounded by a peripheral region of high signal intensity. The underlying formation mechanism may be related to the differential enhancement between the Antoni A zone (cell-dense zone) and the Antoni B zone (mucous-like matrix zone) in schwannomas (20). The “nerve tail sign” serves as a direct imaging marker for neurogenic tumors by directly demonstrates the anatomical continuity between the tumor and the host nerve (21). Additionally, the “nerve effacing sign” may serve as an indicator of malignant tumors with PNS. The solid components of the tumor, characterized by their abundant blood vascularity and contrast agent penetration, exhibit a diffuse signal reduction on CE-MRN. This imaging finding mimics the appearance of affected nerves being incorporated into the tumor mass (22). The novel series of signs offers a new perspective for assessing nerve involvement and tumor features.

It is worth noting that some neurogenic tumors also exhibit the “nerve effacing sign”. We speculate that certain neurogenic tumors may present with marginal enhancement, causing the truncated host nerves at the tumor margins on CE-MRN images. This phenomenon contrasts with the signal reduction observed in non-neurogenic malignancies on CE-MRN images, yet both represent examples of the “negative enhancement” effect of paramagnetic contrast agents on T2-weighted images (23). This study also found that the signal intensity of the affected distal nerves was generally increased compared to the unaffected nerves (24). This could be associated with secondary alterations following nerve injury such as edema, demyelination, and Wallerian degeneration. Previous studies have demonstrated that the signal intensity of injured nerves varies with the progression of different phases. Specifically, during the acute and subacute phases, factors such as edema, demyelination, and Wallerian degeneration may contribute to an increase in signal intensity. In contrast, during the chronic phase, the formation of fibrosis may result in a decrease in signal intensity (25–27).

CE-MRN leverages the T2 shortening effect of paramagnetic contrast agents, and integrates 3D high-resolution T2-weighted inversion recovery sequences to effectively suppress background signals originating from blood vessels, muscles, salivary gland and lymph nodes, thereby significantly enhancing the visualization of nerves (8–10, 28). This imaging technique relies on the existence of the blood-nerve barrier (BNB), which prevents the penetration of contrast agent, thereby maintaining relatively high signal intensity in nerves. However, the processes of tumor tissue neoangiogenesis and BNB disruption facilitate contrast agent penetration, which in turn reduces T2 signal intensity (29), creating high contrast between tumors and nerves. However, this characteristic of CE-MRN can be considered a double-edged sword, as it enhances nerve visibility simultaneously diminishing the delineation of tumor contours. CE-T1WI always clearly displays the solid enhancement components of tumors. The fusion of CE-T1WI and CE-MRN can simultaneously preserve nerve contrast and tumor visibility, facilitating the identification of anatomical relationship between tumors and nerves. This approach not only reduces diagnostic uncertainty but also increases interpreter confidence. In surgical planning for neuro-adjacent tumors, it provides intuitive spatial guidance for optimizing surgical trajectories and minimizing intraoperative nerve injury. For malignancies with neural invasion, the technique enables more precise radiotherapy target delineation, ensuring adequate tumor coverage while protecting critical neural structures. Ultimately, it offers an enhanced visual solution for managing neural tumors.

This study has certain limitations. Firstly, the small sample size and the variety of tumor types could reduce the statistical power. Furthermore, the fusion techniques now require manual registration, which is not only laborious but also subject to subjective bias; thus, In the next phase of our research, We are conducting a prospective cohort study while actively promoting CE-MRN and collecting multi-center cases for external validation, aiming to further evaluate and enhance the general applicability of our research methodology and conclusions. there is a need to develop automated fusion algorithms in the future to enhance efficiency. Future research could combine artificial intelligence algorithms to optimize image registration efficiency, integrate advanced technologies such as DTI and PET-MRI through multimodal integration, and explore multi-parameter quantitative models to improve diagnostic accuracy.

## Conclusion

This study retrospectively analyzed MRI data from patients with neurogenic and non-neurogenic head and neck tumors involving cranial nerves. The results showed distinct imaging characteristics between the two types of tumors on conventional MRI and CE-MRN. Furthermore, the image fusion of CE-T1WI and CE-MRN significantly improved diagnostic confidence and lesion conspicuity, thereby providing a feasible method for simultaneous nerve-tumor visualization in the head and neck.

## Data Availability

The raw data supporting the conclusions of this article will be made available by the authors, without undue reservation.

## Supplementary material

Supplementary Table 1 Inter-observer Consistency of CE-MRN Findings: A Comparison Between Different Scanner Models (Kappa).
