# Supplementary material for: Fusion of contrast-enhanced magnetic resonance neurography and T1-weighted imaging improves simultaneous nerve-tumor visualization in head and neck lesions
Source: Front Oncol. 2025 Oct 22;15:1692893. doi: 10.3389/fonc.2025.1692893 (PMC12586890; doi:10.3389/fonc.2025.1692893)
Supplement: Supplementary Table 1 — Inter-observer Consistency of CE-MRN Findings: A Comparison Between Different Scanner Models (Kappa). [file Table1.docx]

**Supplementary documents**

| **Table S1 Inter-observer Consistency of CE-MRN Findings: A Comparison Between Different Scanner Models (Kappa)** | | |
| --- | --- | --- |
|  | **Siemens（n=15）** | **Philips（n=16）** |
| Enhanced target sign | 1.000 | 1.000 |
| Nerve effacing sign | 1.000 | 1.000 |
| Nerve compressing sign | 1.000 | 0.862 |
| Nerve Tail sign | 0.865 | 0.765 |
| Nerve Signal increase | 1.000 | 1.000 |
| Nerve Signal decrease | 1.000 | 1.000 |
| Nerve hypertrophy | 0.842 | 1.000 |
| Nerve atrophy | 1.000 | 0.862 |
